# Study of the therapeutic effects of an advanced hippotherapy simulator in children with cerebral palsy: a randomised controlled trial

**DOI:** 10.1186/1471-2474-11-71

**Published:** 2010-04-16

**Authors:** Pablo Herrero, Ángel Asensio, Elena García, Álvaro Marco, Barbara Oliván, Alejandro Ibarz, Eva M Gómez-Trullén, Roberto Casas

**Affiliations:** 1 Faculty of Health Sciences. Universidad San Jorge. Autovía A 23 Zaragoza-Huesca, km 510, 50830 Villanueva de Gállego (Zaragoza), Spain; 2 AIDIMO, Belle Epoque 27, 50019 Zaragoza, Spain; 3 Tecnodiscap Group, University of Zaragoza, Maria de Luna 1, 50018 Zaragoza, Spain; 4 Aragonese Health Science Institute, Avda Gomez Laguna n° 25, 50009 Zaragoza, Spain; 5 Dpto de Fisiatría y Enfermería, University of Zaragoza, Domingo Miral s/n 50009 Zaragoza, Spain

## Abstract

**Background:**

Although hippotherapy treatment has been demonstrated to have therapeutic effects on children with cerebral palsy, the samples used in research studies have been very small. In the case of hippotherapy simulators, there are no studies that either recommend or advise against their use in the treatment of children with cerebral palsy. The aim of this randomised clinical study is to analyse the therapeutic effects or the contraindications of the use of a commercial hippotherapy simulator on several important factors relating to children with cerebral palsy such as their motor development, balance control in the sitting posture, hip abduction range of motion and electromyographic activity of adductor musculature.

**Methods/Design:**

The study is a randomised controlled trial. It will be carried out with a sample of 37 children with cerebral palsy divided into two treatment groups. Eligible participants will be randomly allocated to receive either (a) Treatment Group with hippotherapy simulator, maintaining sitting posture, with legs in abduction and rhythmic movement of the simulator or (b) Treatment Group maintaining sitting posture, with legs in abduction and without rhythmic movement of the simulator. Data collection and analysis: all measurements will be carried out by a specially trained blind assessor. To ensure standardization quality of the assessors, an inter-examiner agreement will be worked out at the start of the study. The trial is funded by the Department of Research, Innovation and Development of the Regional Government of Aragon (Official Bulletin of Aragon 23 July 2007), project number PM059/2007.

**Discussion:**

Interest in this project is due to the following factors: Clinical originality (there are no previous studies analysing the effect of simulators on the population group of children with CP, nor any studies using as many variables as this project); Clinical impact (infantile cerebral palsy is a chronic multisystemic condition that affects not only the patient but also the patient's family and their close circle of friends); Practical benefits (the development of an effective treatment is very important for introducing this element into the rehabilitation of these children).

**Trial registration:**

Current Controlled Trials ISRCTN03663478.

## Background

Hippotherapy is a physical treatment strategy in which the movement of a horse is used to improve posture, balance and general development of people with or without motor difficulties. It has been used since the 1960s in Europe and the mid 1970s in the USA for the treatment of cerebral palsy (CP) as well as other neurological pathologies such as multiple sclerosis, traumatic brain injury, learning disabilities, muscular dystrophy and sensorial problems. It is based on current theories of motor control and development, and neurophysiological treatment principles.

Several research studies have been published [1-13] showing the beneficial effects of hippotherapy on patients with disorders of the central nervous system, including infantile CP. Current studies of the beneficial effects of hippotherapy in the treatment of children with CP have a scientific evidence level of 2a [14]. The main studies have been carried out on the basis that the horse's gait provides a precise, rhythmic and repetitive pattern of movement similar to the movements of human walking [15]. Therefore, a patient whose disability has impeded the development of a rhythmic walking pattern can be helped to acquire reciprocal aspects of movement and to improve posture control through the stimulation of normal balance reactions and through repetitive stimuli for posture coordination during the hippotherapy session [16,17].

The therapeutic results obtained with the application of hippotherapy treatments has encouraged research into developing an advanced hippotherapy platform or simulators that "imitate" the movements of a horse, so that this therapy may be more accessible and adaptable to patients.

A detailed study of the work developed in this line of research or associated disciplines enables the following classification to be drawn up of types of equine movement simulators:

Structures of the Karakuri type [18,19] designed to give the sensation of "riding a horse" and "trotting" by means of a combination of forward-backward and up-and-down oscillating movements. It is generally aimed for uses requiring a great sensation of movement rather than fine and precise adjustments [20].

Structures based on hexapods with geometry of parallel platforms, with the upper platform being completely moveable. Hexapods allow a greater degree of moveability and adjustment, ideal for more precise movements at the expense of greater structural complexity.

Technologies with specific mechanisms, such as those developed by Matshushita (Panasonic) or Osim based on the use of eccentric cams and followers (ad-hoc solution).

In any event, the development of mechanical simulation systems for hippotherapy appears to be innovative and not much exploited. There is no diversified work within this research area.

In short, starting from existing studies about the benefits of hippotherapy and taking into account the scarcity of studies carried out with simulators (and none with children with CP) the aim is to develop a study on the effects of the use of a simulator with these children with respect to two actions: remaining in the sitting position (trunk stability) and influence on the hip joint, with the simulator working providing a rhythmic movement imitating a horse walking and with the simulator used simply as a sitting device with no movement.

This study is especially novel for two reasons. First, because the sample of children is substantially greater than that of other published studies concerning traditional hippotherapy, and the study is not limited to less serious cases. Second, because the therapeutic effect of a hippotherapy simulator is to be studied on a population group about whom no published data is available about the use of simulators.

### Objectives

The main objective to be achieved in this project is to study the therapeutic relevance of a hippotherapy simulator for the treatment of children with cerebral palsy (considering four outcome variables, which are: sitting stability, muscle activity in hip adductors, range of hip abduction mobility and global motor development). We will study and divide the effect of the hippotherapy simulator into two aspects:

- Maintenance of a therapeutic stance.

- Rhythmic and repetitive movement produced by the simulator, that is similar to the normal mechanics of the human step.

## Methods

This study has been approved (reference number CP04/06/08) by a Spanish Regional Ethics Committee (CEICA) and consequently the study will be carried out in compliance with the Helsinki Declaration of Human Rights.

Informed consent will be obtained from children's parents or tutors prior to participating in the study.

### Sample

The subjects of the study are children between 4 and 18 years old with CP, who attend schools run by the Education Department of the Government of Aragon (Spain) and whose tutors have signed an informed consent form.

Regarding the sample size, a calculation of statistical power was made previous to the study. The calculation of statistical power was based on a non-published introductory research of the effects of a commercialized hippotherapy simulator for cerebral palsy children. In this study, an improvement of 60% was obtained in cases against 10% of improvement in controls, so improvement probability is considered to be at least double in cases than in controls (odd ratio of 2). In order to assure a power of 0.90 the sample should be 17 in every group (total sample 34). The sample size was considered to be 10% greater due to possible drop-outs (37-38 children).

All CP children that fit the inclusion criteria were selected to participate (37 children). In this study it is hoped to assign at least 18 subjects randomly to each experimental condition.

In cases where tutors or children do not wish to participate, the following variables will be recorded: Sex, age, level of Gross Motor Function Classification System and cause to refuse to participate.

The exclusion criteria are as follows:

- Children who have undergone selective dorsal rhizotomy

- Acute convulsions not controlled by medication

- Allergy to electrode adhesives

- Visual impairment not corrected with glasses

- Those circumstances or associated illnesses that in the judgment of the researcher might interfere with the results or be detrimental to the children.

- Inability to attend intervention sessions or refusal to participate.

### Study design

**Figure 1:**
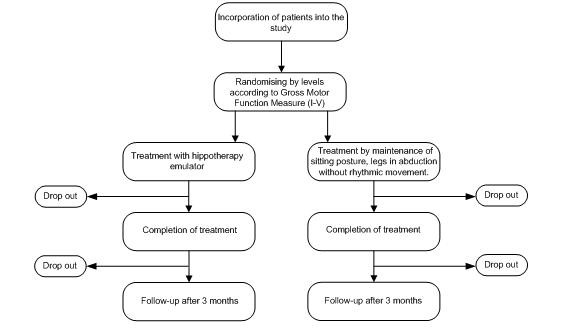
**Randomised controlled trial design**.

The study design is a randomised controlled trial. A scheme of the study is shown in Figure 1.

The patients will be classified into five levels according to the Gross Motor Function Classification System (GMFCS) and subsequently randomly divided into two treatment groups.

1.- Treatment Group with hippotherapy simulator switched on.

2.- Treatment Group with hippotherapy simulator switched off (without rhythmic movement).

The randomising will be centralised and generated by computer according to stratified groups to ensure a similar distribution of the possible confounding variables in both groups. The assignation sequence will be concealed.

Ten treatment sessions (one weekly) will be given in each modality, in which the children will do different activities proposed by the physiotherapist according to their motor possibilities.

In the treatment sessions with the hippotherapy simulator switched on, the children will maintain the sitting position for 15 minutes on the simulator, with an active extension of the trunk, stabilization of the pelvis and abduction of hips while the simulator produces a rhythmic and repetitive movement that is similar to a walking horse. In the treatment sessions with the simulator switched off (without providing the rhythmic movement), the children will maintain the active extension of the trunk, stabilization of the pelvis and abduction of hips but they won't benefit from the rhythmic effects produced by the hippotherapy simulator. That is, the children who sit on the simulator that is switched off will receive the same treatment except that they won't receive rhythmic movement from the simulator (it would be similar to sitting on a barrel or another device in which they maintain this sitting position, similar to other studies published^7^).

Children not included in the group with the simulator switched on will be offered the possibility to receive ten more treatments with the simulator switched on once the research has concluded.

### Study variables

The hippotherapy simulator used for this study is "Core Trainer Exercise Equipment, JOBA" produced by Matshuita (Panasonic), and allows working at 9 speeds and 3 predefined programmes (basic conditioning, waist, hips).

This simulator was designed to be used in fitness centres. To make it easier to use the system offers three automated training routines:

WORKOUT (Side-To-Side)

WAIST (Forward Tilt)

HIPS (Backward Tilt)

These training routines can automatically modify the speed and tilt of each one of the training sub phases

When planning a work session with the JOBA commercial Simulator, the time, tilt and speed adjustment allows for unequivocally defining the test parameters. Therefore using the same Simulator and knowing these parameters, the repeatability of the test is totally guaranteed. In the case of our study, we have listed the exact programme that was used, which is Side-To-Side WORKOUT.

The study variables will be measured with the instruments showed in table 1 (see table 1).

**Table 1:** Study variables and instruments for measurement.

STUDY VARIABLES	INSTRUMENTS
Sitting balance	SAS (Sitting Assessment Scale) [21]

Muscular activity in hip adductors	Surface Electromyography (EMG)

Hip abduction range of motion	Electronic inclinometer and traditional goniometer

Gross motor function	Gross Motor Function Measure (GMFM)

There are four outcome variables:

- Sitting balance

- Measurement of hip abduction range of motion

- Electromyographic activity in adductors

- Global motor development

The outcome variables will be measured at three different times during the research. A first measurement will be made at the beginning of the study, a second one when the ten treatment sessions have concluded, and a third measurement after the 3-month follow-up period. Moreover, two of these outcome variables (hip abduction range of motion and Electromyographic activity in adductors) will also be measured before and after each treatment session.

Control of confounding variables (independent variables) will be carried out on each and everyone of the outcome variables studied. The confounding variables considered to be studied will be those listed below:

- Sociodemographic variables: sex, age and physical activity.

- Clinical variables: oral anti-spascity medication treatment, other medical treatments that could interfere with the hippotherapy treatment (antiepileptic drugs, etc.), medical diagnosis, previous experience in hippotherapy, off-trial hippotherapy treatments, surgical interventions, botulinum toxin injections and use of ortheses.

A questionnaire will be completed at the beginning of the study that collects the confounding variables previously described. Monitoring will be carried out throughout the duration of the study.

Data collection and analysis: all measurements will be carried out by a specially trained blind method consultant. It is a triple blind trial because patients, researchers and statisticians were unaware of the group the subjects of the study belonged to. To ensure standardization quality of the consultants, an inter-examiner agreement will be worked out at the start of the study.

### Statistical analysis

The data will be entered and analyzed with SPSS 14.0, and the following statistical procedure will be undertaken:

1. Descriptive analysis of defined variables.

2. Mean comparison tests for quantitative variables and comparison of proportions for qualitative data, among independent variables to check the comparability of the groups.

3. Mean comparison tests for each outcome variable according to the treatment group (differences pre-post treatment in both treatment groups as well as differences between them)

4. Multivariate analysis to determine the influence of independent variables in the evolution of each outcome variable.

A descriptive analysis of drop outs will also be made in order to determine if there are correlating patterns.

## Discussion

The interest in this project is due to the following factors:

- Clinical originality: there is no previous studies that analyse the effect of simulators on the population group of children with CP, nor that use as many variables as this project. Furthermore, the sample under study is noticeably larger than others in the literature concerning traditional hippotherapy, and includes a greater range in terms of the degree to which patients are affected.

- Clinical impact: infantile cerebral palsy is a chronic multisystemic condition that affects not only the patient but also the patient's family and their close circle of friends. Affected children receive a large number of treatments from a multi-disciplinary team (physiotherapists, special needs teachers, speech therapists, occupational therapists, etc). It is therefore important to make advances in the clinical treatment of these children (especially through techniques that could be considered as recreational) to improve their quality of life.

- Practical benefits: the development of an effective treatment is very important for introducing this element into the rehabilitation of these children. The effectiveness of hippotherapy has already been demonstrated but in many cases it is not possible to put it into practice for various reasons (fear, difficulty of mounting a horse, climate, financial considerations, etc). In these circumstances, if the use of simulators can be shown to be therapeutically effective, it would benefit a considerable number of patients.

- Increase in technological experience: the knowledge acquired during the development of the project would lead to further lines of research in very important areas such as mobility, automated physiotherapy, and ergonomic positioning measures.

- Social advantages: the opportunity of offering new evidence-based treatments for infantile cerebral palsy is of great interest at a social level not only for the benefits to be derived from the therapy but also for the recreational character of the activity and the possibility of combining with other types of therapy.

## Competing interests

The authors declare that they have no competing interests.

## Authors' contributions

All authors contributed equally to this work. Aspects related to cerebral palsy were carried out by PH, EG, BO and EMG. Technical aspects such as engineering devices were explained mainly by AA, AM, AI and RC.PH designed the study protocol, carried out the literature review and coordinated the clinical part of the study. EG completed the literature review, helped to coordinate the clinical part and carried out all the process regarding giving information and selecting patients. BO carried out all the procedures to apply for Ethical Approval, assessed study design and helped with the selection of patients.

EMG helped with the design of the study protocol and reviewed all methodological and statistical parts of the protocol. AA completed the literature review of technical devices and provided technical protocol. AI carried out the literature review of technical devices and provided technical protocol. AM reviewed technical protocol and supported the clinical team with all technical needs related to the hippotherapy simulator. RC coordinated all the technical work. All authors read and approved the final manuscript

## Pre-publication history

The pre-publication history for this paper can be accessed here:

http://www.biomedcentral.com/1471-2474/11/71/prepub
